# Helen B. Taussig (1898-1986)

**Published:** 2016-09

**Authors:** J Van Robays

**Affiliations:** Department of Pathology, ZOL, Campus St Jan, Schiepse Bos 6, 3600 Genk, Belgium.

**Figure:**
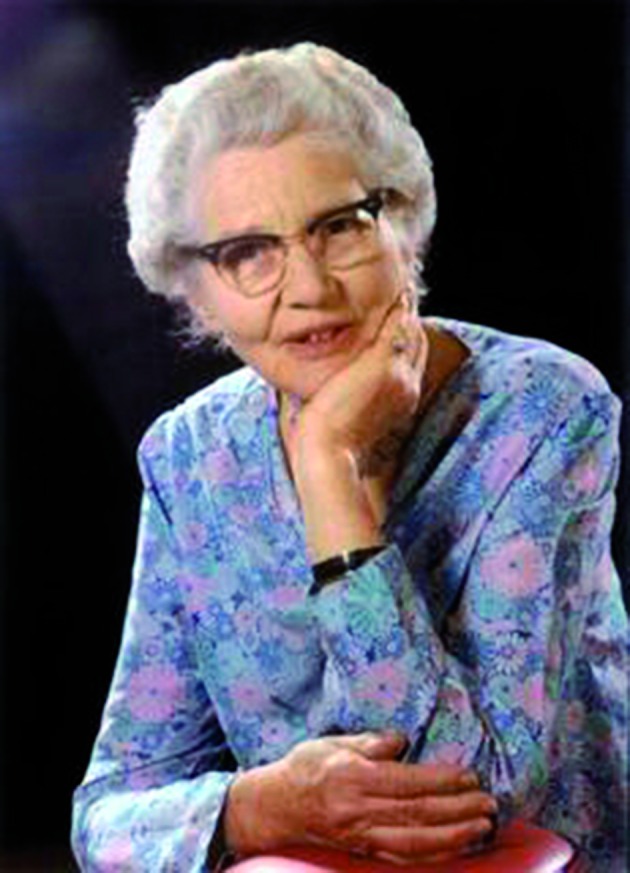


## Introduction

Together with Alfred Blalock, Helen Taussig rose to fame because of the Blalock-Taussig shunt. This is a surgical procedure in children who suffer from hypoxia because of a congenital cardiac malformation. Because of this affliction, they are blue. Less known is the fact that dr. Taussig also played a crucial role in the prohibition of thalidomide in the USA, the drug that had been responsible for the Softenon tragedy in Europe.

## Dyslectic and deaf

Helen Taussig was born on the 24th of May, 1898, in Cambridge, Massachusetts, as the youngest of four children. Her father, Frank Taussig, was a professor in Economy at Harvard University. Her mother had been one of the first female graduates at the Radcliffe College, where she had studied biology and zoology. However, she died of tuberculosis when Helen was only 11 years old. Helen’s grandfather, William Taussig, had been a teacher in a school for blind children. As a tribute to his services, a school was named after him: the William Taussig School for Handicapped Children. As Helen herself said, she descended “from a direct line of teachers, and an indirect line of doctors”.

In the Cambridge School for Girls, she didn’t exactly get top grades. After an innocent flu, she had gotten a middle ear infection, which rendered her half deaf. Furthermore, she was having trouble reading. When her father discovered her dyslexia, he helped her spelling words and reading texts. A successful endeavour, as it turned out, as Helen followed into her mother’s footsteps and took up studies at the Radcliffe College in 1917. Here, she attained good grades and even became the college’s tennis champion. Two years later, she left for Berkeley University in California, where she got her baccalaureate degree in 1921. Afterwards, she wanted to continue her education at Harvard University, but it wasn’t until 1945 that this male bastion allowed women to enter.

## From pillar to post

Her father thought it wiser for a girl to work for the Department of Public Health than to become a physician anyway, so he sent her to the Harvard School of Public Health. There, Helen soon got into trouble with an unfriendly dean. She was allowed to follow the lectures, but it was made clear that as a woman, she shouldn’t expect to obtain a degree. To study for four years without the prospect of a proper degree seemed a bit too ridiculous to Helen, so she decided to go to Boston University’s Medical School instead. There she could study biology and anatomy, even if it was still separated from the male students. Especially the inspection of anatomical pictures had to take place in a secluded room. Whilst writing a thesis on the muscular bundles of a cow’s heart, Helen acquired an interest in the human heart. She delivered a beautiful thesis, and as a consequence her professor advised her to take up studies at the Johns Hopkins Medical School in Baltimore. It was one of the few American universities at that time that allowed women to enter. After obtaining her M.D. in 1927, Helen desperately wanted to specialize in Internal Medicine. However, there was only one position for a woman in that discipline available, and it was already taken. Helen thus had to change her mind to Paediatrics, a decision which turned out to have great consequences, not just for her own career but for the medical world in general. She would eventually give an enormous boost to a discipline that was at that time still in its infancy: paediatric cardiology. To detect a cardiac malformation, a physician around the year 1930 needed three things: knowledge of the normal and abnormal heart sounds, a stethoscope and a good ear. Helen was unfortunately no longer in the possession of the latter. Her deafness continued to worsen over the years. Initially, she could get by with the help of a hearing aid and an auditory amplifier on her stethoscope. But in due time she learned to lip read and to “listen with her fingertips” to her young patients’ heart. She eventually got so proficient in “listening with her fingertips”, that she wasn’t inferior to a paediatrician with normal hearing and a normal stethoscope when it came to detecting heart sounds.

**Fig. 2A:**
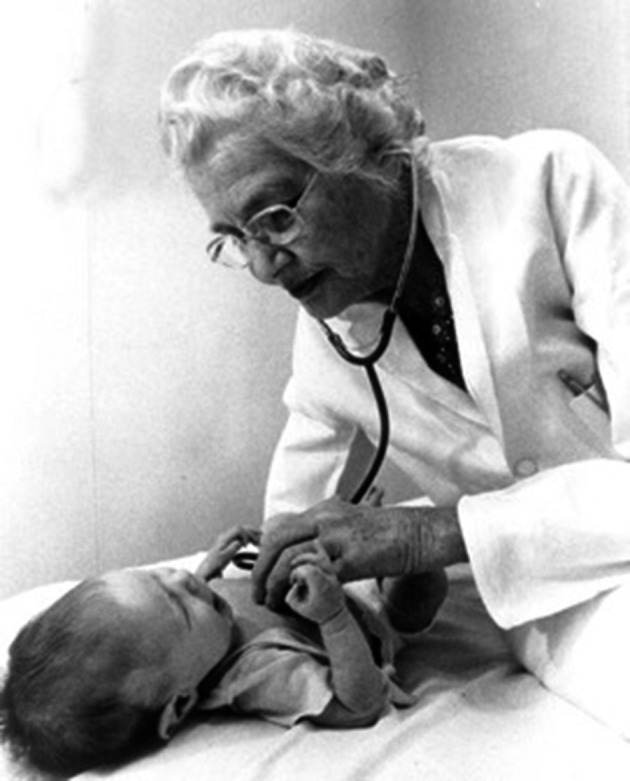
— Dr Taussig using the stethoscope, before the hearing problem

**Fig. 2B:**
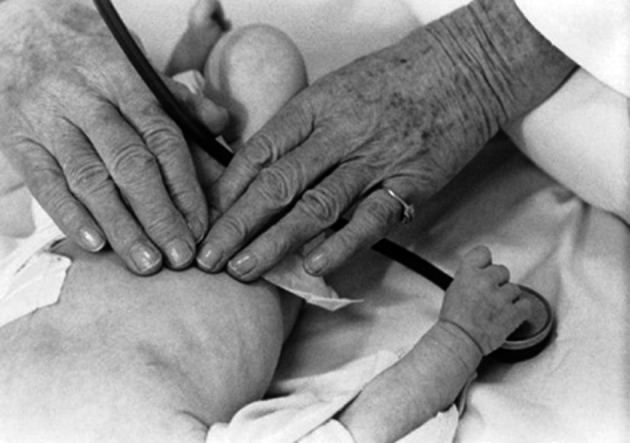
— Dr Taussig using her fingertips to examine her young patients’ heart

## Blue baby syndrome

In her paediatric practice, dr. Taussig frequently saw babies who looked blue during and right after the delivery. After every sip at their mother’s breast, they had to gasp for air. They had a congenital malformation, most frequently a tetralogy of Fallot. Because of a narrow pulmonary artery, too little blood can flow to the lungs, where it is normally abundantly provided with oxygen. But why was it, she wondered, that some of these “blue babies” died soon after birth, while others survived for months or even years? Was there a pattern here, a system? A special vessel caught her attention: the *ductus arteriosus*. This is a short connection between the pulmonary artery and the aorta. For the foetus in utero, it is a necessary shunt between the pulmonary and systemic circulatory system. After birth, it is no longer needed, and becomes even detrimental. When the *ductus arteriosus* doesn’t close spontaneously, a surgical intervention is needed. The vascular surgeon most skilled in closing the *ductus*, was dr. Robert Edward Gross from Boston.

In the meantime, Helen made another important discovery. The blue babies whose *ductus arteriosus* stayed patent after birth, did remarkably better than those whose *ductus* closed spontaneously. They looked less blue and survived for longer. So it must be important, she reasoned, to keep that shunt patent. And if it were to close spontaneously, to replace it by an artificial shunt. With this in mind, she went to the man who had closed so many patent *ducti arteriosi* successfully, and was known far beyond Boston as the “ductus surgeon”. But when Helen came with her proposal, dr. Gross had his doubts: “I have enough trouble closing a patent ductus, without creating one!” A remark he would later on come to regret and consider the most idiotic thing he had ever said.

## Alfred Blalock (1899 – 1964)

After this rejection, Helen went to a heart surgeon, who had been appointed recently at Johns Hopkins because of his experience and expertise. At the Vanderbilt University in Nashville, dr. Blalock had conducted many experiments, among which the creation of an animal model to study pulmonary hypertension. Helen Taussig asked him if he would be able to create an artificial shunt to give her “blue babies” a chance to life. Alfred Blalock didn’t turn this proposal down right away, although he did need some time to think about it. Technically speaking, such a shunt was surely possible, but he would prefer to test it first on an animal model. Together with his technician, Vivien Thomas, he constructed an end-to-side connection between the subclavian artery and the pulmonary artery. And it worked! Not just in technical terms, but also on a physiological level. An abundance of oxygen flew through the venous system. When the “Blalock-Taussig shunt”, as this connection was called, worked perfectly in dogs, the time had come to test it for the first time on a blue baby.

**Fig. 3:**
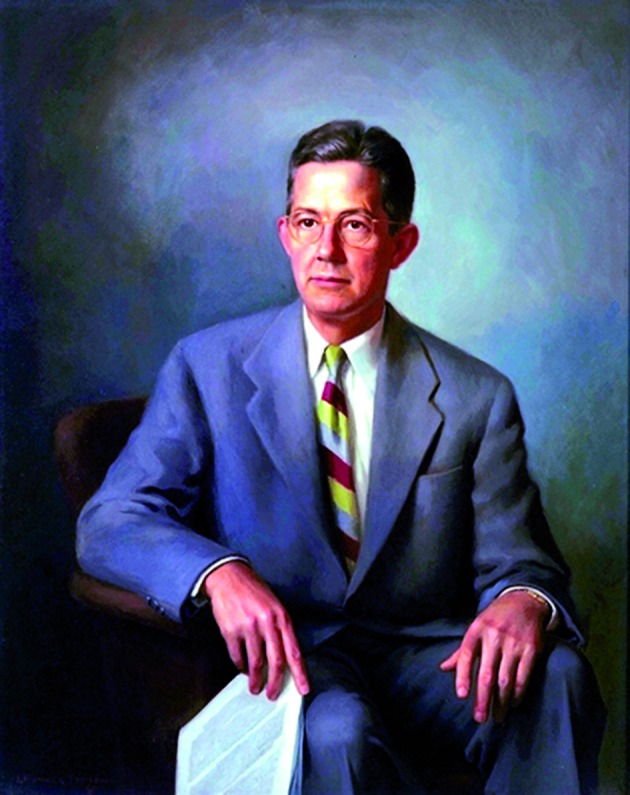
— Alfred Blalock

In the beginning of November 1944, a very ill child arrived at the emergency department of Johns Hopkins Hospital. She looked purple-blue and could hardly drink a sip without gasping for air. At the age of 15 months, little Eileen Saxon weighed only 5 kg. With her sensitive fingertips, Helen Taussig diagnosed a Tetralogy of Fallot with severe pulmonary hypertension. If nothing was done, little Eileen was condemned to a certain death. On the 9th of November, 1944, she was brought to theatre and anesthetized. Backed up by his technician, Vivien Thomas, dr. Blalock cut the artery going to the arm, and grafted it sideways on the pulmonary artery. Helen was watching from a distance, biting her nails and wholeheartedly hoping the operation would succeed. Luckily, it worked. After the surgery, Eileen changed colour immediately, from a sickly blue to a blushing pink complexion. Without having to gasp for air, she could drink milk again and quickly gained a few kilograms. Two months later she could leave Johns Hopkins Hospital, alive and kicking.

## Vivien Thomas (1910 – 1985)

Beyond the scenes of this success story, there was another man who has by now been completely forgotten. As the grandson of an imported slave, Vivien Thomas had made it from a jack-of-all-trades to dr. Blalock’s medical technician, even without having a degree. Ever since the time dr. Blalock experimented on dogs at the Vanderbilt Institute, Thomas had been his factotum and right hand. To honour his – non-negligible – contribution to the “Blalock-Taussig shunt”, it should have actually been called the “Blalock-Thomas-Taussig” shunt. But Vivien Thomas was a person of colour, and in the America of those days, therefore on an ever lower social rank than women.

**Fig. 4:**
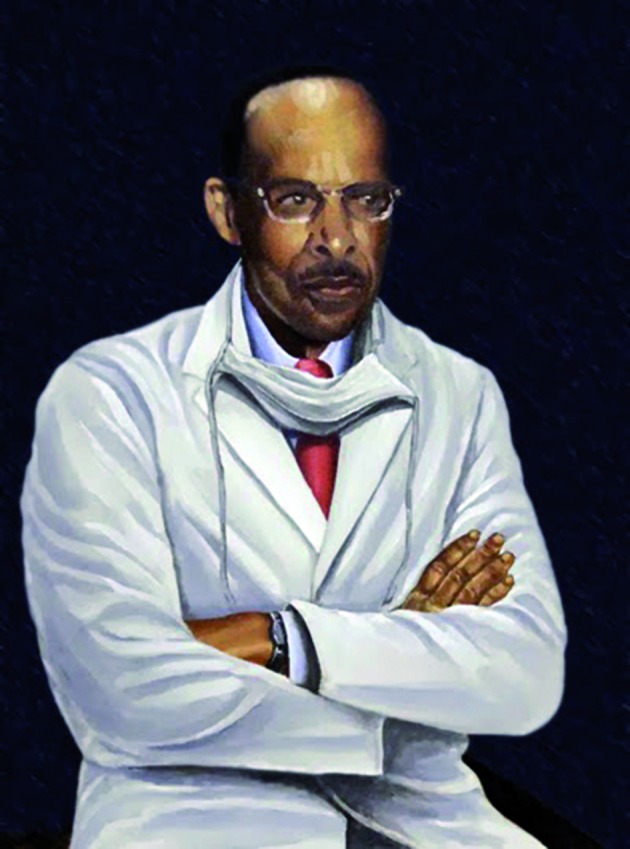
— Vivien Thomas, Dr Blalock’s medical technician

## Worldwide succes

After the third successful operation, Alfred Blalock and Helen Taussig published their results in The Journal of the American Medical Association. The Blalock-Taussig shunt became world famous overnight. From everywhere in America and Europe, they were asked to further explain this surgical procedure in various lectures and conferences. Soon the story of this miraculous cure also made its appearance in non-medical magazines. Thereafter, many parents of blue babies went to Johns Hopkins Hospital to have their baby examined by dr. Taussig and operated on by dr. Blalock. For medical residents and aspiring surgeons as well, the children’s cardiology centre in Baltimore became the place to be. Many stuck around and gradually learned the technique themselves. By the end of 1951, the surgical team had given 1.037 blue babies a chance to life. Mortality was only 5%.

In 1947 Helen Taussig summarized her years of anatomical research and great clinical experience into a book: “Congenital malformations of the heart”. It soon became the standard work for paediatric cardiology and surgery, at the time still in its infancy. With her insight into the hemodynamics of cyanosis, she would later be referred to as “the mother of paediatric cardiology”.

**Fig. 5:**
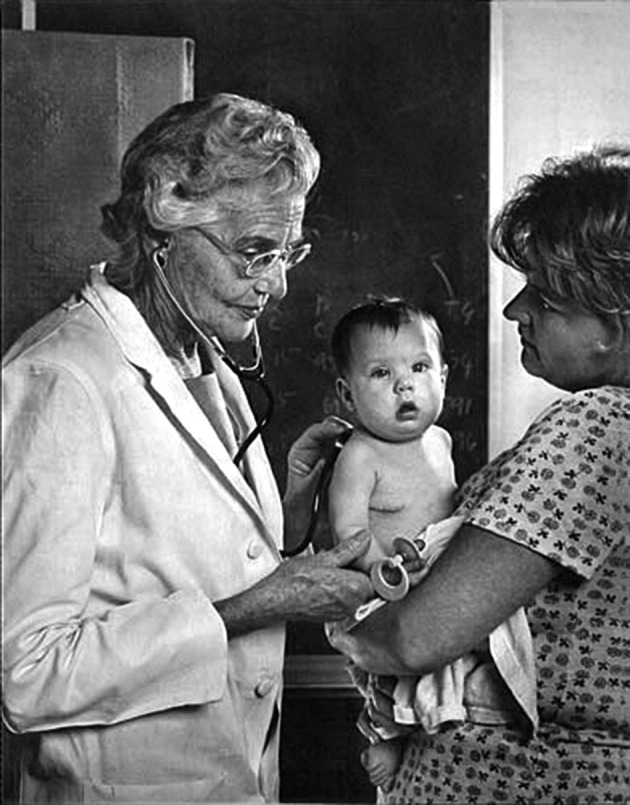
— Helen Taussig examining a blue baby

## The near-tragedy of Softenon in the USA

Around 1960, many babies were born in Germany, the Netherlands and Belgium who suffered from a till then rare disease: phocomelia. The newborns didn’t have any arms, and their hands were attached straight to their shoulders. Immediately the search began for the cause of this horrible and mysterious disfiguration. Was it a genetic condition? Was it related to environmental pollution? Nuclear radiation? Nuclear experiments? All possible trains of thought were put forth until in November 1961, the German physician-geneticist Widukind Lenz was the first to make the connection between phocomelia and the use of the drug thalidomide.

Thalidomide was a drug initially intended as anti- epileptic drug, but which unfortunately didn’t have any effect against epileptic seizures. It did induce somnolence, however. And so by the end of 1957, it was marketed as sleeping medication and sedatives by the German pharmaceutical Chemie Grunenthal. It quickly became the most popular sleeping pill in Europe and was also promoted as an anti-emetic in pregnant women suffering from morning sickness. But when pregnant women take the drug between day 35 and 49 of their pregnancy, when the embryo’s arms and legs are formed, this foetal development is blocked.

Helen Taussig was told of this European Softenon tragedy by one of her students in January 1962. She immediately decided to get to the bottom of this and crossed the Atlantic Ocean. In various European cities, she went to examine those arm- or legless babies and figure out their medical history. Her conclusion matched that of dr. Widikund Lenz; the sedative thalidomide (Softenon) was the culprit. As soon as possible, she flew back to the States to stop the pending approval of this drug by the Food and Drug Administration (FDA). Convinced that Softenon could cause horrific malformations in embryos, she started a grand campaign. Helen gave lectures for the American College of Physicians and the American Senate. She wrote articles in various non-medical journals and everywhere she took a stand to warn pregnant women against the use of thalidomide, and any other drug during pregnancy for that matter. Because often women carelessly take medication at a time that they’re not even aware of their pregnancy yet, resulting in at times catastrophical consequences for the developing foetus in their womb.

With these arguments, Helen Taussig could eventually convince the American senate and the FDA. Despite the powerful pharmaceutical lobby, Thalidomide did not enter the American market. In addition to the thousands of “blue babies” she had given a new chance to life, together with Blalock and Thomas, she probably saved tens of thousands more from the Softenon-malformations.

Moreover, her campaign had far-reaching consequences. In 1963, a new law was voted to test new drugs more rigorously, before bringing them on the market.

## Last pain

When Helen reached the age of retirement in 1963, she didn’t think about slowing down. Testimony to this is the fact that of the hundred of articles she wrote throughout her life, forty were published after her retirement. There was no lack of recognition and honorary doctorates in the years to come. At the age of 67, she even became the first female president of the most renowned cardiologic institute in the USA: the American Heart Association. This (belated) recognition brought her much joy. Despite the many handicaps she had faced throughout her life, deafness, dyslexia, her mother’s early demise and sexist discrimination, she had been able to realize a lot. About the only thing that had hurt her, she wrote the following: “Over the years I’ve gotten recognition for what I did, but I didn’t at the time. It hurt for a while. It hurt when dr. Blalock was elected to the National Academy of Arts and Sciences, and I didn’t even get promoted from an assistant to associate professor.”

At the end of the seventies, Helen moved to Pennsylvania. Three days before her 88th birthday, on the 21st of May 1986, she died in a car crash. As had happened to her grandfather, an institute was subsequently named after her. As a reminder to the woman who had solved the mystery of the “blue babies” in Johns Hopkins Hospital, it was named: Helen B. Taussig Children’s Pediatric Cardiac Center.

